# How to distinguish between different cell lineages sharing common markers using combinations of double in-situ-hybridization and immunostaining in avian embryos: CXCR4-positive mesodermal and neural crest-derived cells

**DOI:** 10.1007/s00418-020-01920-7

**Published:** 2020-10-10

**Authors:** Imadeldin Yahya, Marion Böing, Beate Brand-Saberi, Gabriela Morosan-Puopolo

**Affiliations:** 1 Department of Anatomy and Molecular Embryology, Institute of Anatomy, Ruhr University Bochum, Bochum, Germany; 2 Department of Anatomy, Faculty of Veterinary Medicine, University of Khartoum, Khartoum, Sudan

**Keywords:** Chicken embryo, Double whole-mount ISH, Immunostaining, CXCR4, NCCs, Mesodermal cells

## Abstract

Cell migration plays a crucial role in early embryonic development. The chemokine receptor CXCR4 has been reported to guide migration of neural crest cells (NCCs) to form the dorsal root ganglia (DRG) and sympathetic ganglia (SG). CXCR4 also plays an important part during the formation of limb and cloacal muscles. NCCs migration and muscle formation during embryonic development are usually considered separately, although both cell lineages migrate in close neighbourhood and have markers in common. In this study, we present a new method for the simultaneous detection of CXCR4, mesodermal markers and NCCs markers during chicken embryo developmental stages HH18–HH25 by combining double whole-mount in situ hybridization (ISH) and immunostaining on floating vibratome sections. The simultaneous detection of CXCR4 and markers for the mesodermal and neural crest cells in multiple labelling allowed us to compare complex gene expression patterns and it could be easily used for a wide range of gene expression pattern analyses of other chicken embryonic tissues. All steps of the procedure, including the preparation of probes and embryos, prehybridization, hybridization, visualization of the double labelled transcripts and immunostaining, are described in detail.

**Electronic supplementary material:**

The online version of this article (10.1007/s00418-020-01920-7) contains supplementary material, which is available to authorized users.

## Introduction

The neural crest is a migratory embryonic cell population that gives rise to numerous structures in vertebrates (Bronner 2012). In the trunk, these cells migrate to form amongst others sensory and sympathetic ganglia and adrenal medulla (Aoki et al. 2003; Bronner 2012; Cheng et al. 2000). Although the HNK1 epitope is a well-known, reliable and easy marker for chicken NCCs (Giovannone et al. 2015), Sox10 is one of the best universal markers to use when studying NCCs (Cheng et al. 2000). In chicken, Sox10 is detected in migrating NCCs shortly after Slug, but is diminished as cells undergo neuronal differentiation in ganglia of the peripheral nervous system (Cheng et al. 2000). The transcription factor activating protein (Ap2α) has been identified as the regulatory factor for face and limb development (Shen et al. 1997). Chicken Ap2α is expressed in the distal limb bud mesenchyme and its expression is associated with limb bud outgrowth (Shen et al. 1997).

All of the skeletal muscles present in the trunk and limb develop from the dermomyotome, a derivative of the somitic mesoderm (Nogueira et al. 2015; Vasyutina et al. 2005). The dermomyotomes start to express the premyogenic genes (Pax3 and Pax7) as soon as they form. These genes continue to be expressed in the dermomyotome and migratory muscle precursor cells (Nogueira et al. 2015). After delamination from the dermomyotome, myogenic progenitors that form the epaxial and hypaxial muscles continue to proliferate and start to express MyoD and Myf5 (Buckingham 2017; Kablar et al. 1997). At limb level, myogenic progenitor cells migrate as single cells into the limb bud to contribute to the muscles of the limb (Buckingham 2017; Nogueira et al. 2015; Vasyutina et al. 2005). Migration of muscle precursor cells require signals that allow the cells to stay motile and find their final destinations (Vasyutina et al. 2005).

Cell migration is a crucial event in the development and maintenance of multicellular organisms. Chemokine receptors represent a critical class of molecules associated with the control of cell migration. The chemokine receptor CXCR4 has been reported to play important roles during the formation of limb and cloacal muscles (Hunger et al. 2012; Rehimi et al. 2010; Vasyutina et al. 2005; Yusuf et al. 2005). Furthermore, CXCR4 is crucial for migration of sympathetic ganglia progenitor cells (Kasemeier-Kulesa et al. 2010).

The use of the chicken embryo model for developmental experiments belongs to the traditional methods of cell lineage studies. Since decades, the low-cost maintenance, easy accessibility in ovo, and especially the possibility to carry out experimental modifications in the course of embryonic development were the main attractions of this model in research (Tolosa et al. 2013). However, although several primary or secondary antibodies that are necessary in detecting different cell types in the human and mouse are identified, several antibodies in chicken remain unavailable. The lack of specific primary antibodies for immunohistochemistry prevents scientists from detecting several genes simultaneously in the same section (Jolly et al. 2016).

ISH techniques allow a probe (labelled complementary RNA or DNA strand) to localize a specific RNA or DNA sequence in the whole-mount or tissue sections (Lee et al. 2013). Even though the existence of several methods to detect the expression of genes (permanent cell labelling and clonal analysis), ISH remains one of the most widely used methods to study gene expression (Duncan et al. 2015). In the past decade, most studies relied upon combining ISH with immunohistochemistry in mouse brain and kidney sections (Chaudhuri et al. 2013; Georgas et al. 2008; Grabinski et al. 2015; Jolly et al. 2016; Kumar et al. 2012; Lopez 2014; Pineau et al. 2006). Currently, no methods based on the combined double whole-mount ISH with immunostaining have been described for chicken embryos, we set out to develop an optimized way to detect the expression pattern of at least three genes at the same time in the NCCs and mesodermal cells of the developing forelimb.

## Materials and methods

### Chicken embryos

Freshly laid fertilised chicken eggs were cleaned with 70% alcohol and incubated at 37.8 °C with 80% humidity until appropriate developmental stages. The dissection tools were sterilized in a dry oven at 180 °C for 3 h. Stereo microscope and lab bench surfaces were rinsed with 70% ethanol. The embryos ranging from stage HH18–HH25 according to the Hamburger and Hamilton (1992) staging system were dissected in PBS and fixed overnight in PFA 4% in PBS at 4 ºC after removing the extra-embryonic membranes.

### Probe preparation

Tissue samples from chicken embryos were collected and total RNA from homogenated tissue samples were extracted using TRIzol Reagent (Invitrogen). The RNA pellet was dissolved in RNase-free water. The RNA samples were checked for concentration and purity. Specific primers (Primer-Blast, NCBI) were designed in the ORF of desired gene with a product length between 500 and 800 bp approximately. Specific sequences of genes of interest (e.g. Ap2α) were obtained by PCR using Phusion-DNA-Polymerase (Invitrogen) with a pair of gene specific primers (Table 1). The generated blunt-ended PCR product was ligated into pJet vector according to manufactures protocol (CloneJET PCR Cloning Kit, Thermo Scientific) and amplified by bacterial culture. XbaI/XhoI flanked sequence from pJET was cloned into pBluescript II KS + vector and confirmed by sequencing. For linearization, the plasmid was digested with restriction enzymes (Table 2) for 3 h at room temperature. The labelling of the RNA probes was performed by incubating 1 µg linearized plasmid with 4 µl DTT (0.1 M), 2 µl RNA labelling mix (10X DIG or FITC, Roche), 0.5 µl RNase inhibitor, 2 µl transcription buffer (10x, Roche) and 1 µl T3 or T7 RNA polymerase (10 µ/µl, Roche) in a total volume of 20 µl DEPC-H_2_O at 37 °C overnight. The sense RNA probe for Ap2α was synthesized as control (Table 2). RNA probes were extracted using ammonium acetate precipitation (30 µl 10 M ammonium acetate, 250 µl cold (− 20 °C) EtOH, 20 µl transcription mix). The mixture was frozen in liquid nitrogen and centrifuged for 10 min at 13,000 rpm at room temperature. The pellet was washed with 500 µl 80% EtOH and centrifuged for 10 min at 13,000 rpm at room temperature. The probe pellet was dried under the hood and stored at − 20 °C (the quality and final concentration of the probe may be checked again on a gel).

**Table 1:** Ap2α (Transcription factor Activating Enhancer Binding Protein 2 Alpha) primers

	Sequence (5'->3')	Template strand	Length	Start	Stop	Tm	GC%
Forward primer	GCTCTGGAAGCTGACGGATAA	Plus	21	94	114	59.86	52.38
Reverse primer	AGGAGACGGCGTTGTTGTTA	Minus	20	665	646	59.61	50.00
Product length	572						
Target	NM_205094.1 Gallus gallus transcription factor Ap-2 alpha (TFAP2A), mRNA

**Table 2:** Enzymes to linearize pBluescript II KS+-Ap2α

Probes	Restriction enzymes	Promotor/polymerase
Anti-sense	NotI or XbaI	T3
Sense	XhoI	T7

### Antibodies

The conditions and the source of antibodies used for double whole-mount ISH and immunostaining were summarized in Table 3.

**Table 3:** The conditions and the source of antibodies

Antibody	ID	Application	Dilution	Biological source	Manufacturer
Desmin	M0760	Immunostaining	1:300	Mouse monoclonal	Dako
Dig-AP	11093274910	In situ hybridization	1:2000	Sheep polyclonal	Sigma
FITC−AP	A1812	In situ hybridization	1:2000	Mouse monoclonal	Sigma
HNK1	AB_2314644	Immunostaining	1:100	Mouse monoclonal	Hybridoma Bank
Nkx2.2	AB_531794	Immunostaining	1:500	Mouse monoclonal	Hybridoma Bank
Anti-mouse IgG HRP	Po447	Immunostaining	1:1000	Goat polyclonal	Dako

FITC fluorescein isothiocyanate, Dig digoxigenin, Nkx2.2 NK2 homeobox 2, HNK-1 N-linked carbohydrate marker, Anti-mouse IgG HRP Goat anti-mouse IgG horse radish peroxidase.

### Double whole-mount ISH

One day before prehybridization step, the selected embryos were transferred in a sterile 24 well plates in 4% PFA in PBS. The probes and the stages of the embryos were mentioned on the cover of the plate. The fixed embryos were washed with PBST 2 times for 5 min each and digested in proteinase K solution (20 μg/ml) for 15 min at room-temperature (on shaker). After digestion, the embryos were then fixed in 4% PFA for 20 min and washed with PBST 2 times for 5 min each at room-temperature (on shaker). For hybridization**,** the embryos were incubated for 2 h in hybridization buffer at 65 °C (hybridization oven), and then incubated overnight in hybridization buffer + DIG and FITC probes (1 μg/ml for each probe) at 65 °C (hybridization oven). Antisense RNA DIG or FITC probes against CXCR4, Myf5, Pax3, Sox10, Ap2α and Slug were used. An AP2α sense probe was used as a negative control. The embryos were then washed with posthybridization buffer (1) two times for 30 min at 65 °C (hybridization oven). After hybridization**,** these embryos were washed with posthybridization buffer (2) two times for 30 min each 65 °C (hybridization oven), and then washed with 1 × KTBT 3 times for 10 min each at RT (on shaker). For blocking, the embryos were incubated with lamb serum solution 20% in KTBT for 2 h at RT (on shaker). The embryos were then incubated overnight with Anti-digoxigenin AP (diluted in lamb serum solution 1:2000, Sigma) at 4 °C (on shaker). The embryos were washed with 1 × KTBT six times for 1 h each at RT and washed overnight at 4 °C (on shaker). For DIG staining, the embryos were washed with Alkaline phosphatase (AP) buffer three times for 10 min each wash at RT (on shaker), and then incubated in the dark with staining solution (20 μl/ml NBT/BCIP in AP buffer). When staining was finished, the embryos were washed with AP buffer (pH 9.5) three times for 15 min. Before the FITC detection round, the DIG-AP conjugate was inactivated by incubating the embryos in KTBT for 30 min at 65 °C (hybridization oven) or in 0.1 M glycine–HCl (pH 2.2) for 10 min at room-temperature (on shaker). Afterwards, the embryos were washed with KTBT two times for 30 min each at RT (on shaker) and blocked in lamb serum solution 20% in KTBT for 2 h at RT (on shaker). After blocking, the embryos were incubated overnight with Anti-FITC AP (diluted in lamb serum solution 1:2000, Sigma) at 4 °C (on shaker). The embryos were washed with 1 × KTBT six times for1 h each at RT and washed overnight at 4 °C (on shaker). For FITC staining, the embryos were washed with AP buffer three times for 10 min each wash at RT (on shaker). As a substrate, Fast Red diluted (5: 10) in AP Buffer pH 8 was used. When staining was finished, the embryos were washed with AP buffer (pH 8) three times for 15 min each at RT (on shaker). To reduce the background, the embryos were washed with TBST three times for 30 min each at RT, and then washed with PBST two times for 10 min each wash at RT (on shaker). Finally, the embryos were fixed in 4% PFA overnight at 4 °C. Selected double stained embryos are processed for immunostaining or may be stored in 4% PFA in PBS for several months.

### Immunostaining

After double whole-mount ISH has been completed, the embryos were transferred to a small Petri dish and washed three times in 1 × PBS for 10 min each to remove the PFA. Under the stereomicroscope, the heads were removed with blunt forceps. 3 g agarose in 100 ml of distilled water was heated on a microwave set to 800 watts until dissolved. The heated agarose was poured into 35 × 10-mm Petri dish. The embryo trunk regions were transferred by a perforated spoon into 35 × 10-mm Petri dish. The tissues were embedded by adjusting the plane of sections under stereo microscope with blunt forceps. Next, the tissue blocks were cooled to room temperature until the agarose solidified. The agarose blocks were trimmed to expose the tissue surface. The blocks were glued to metal chuck using Pattex glue. The blocks were cut in distilled H_2_O at 50 µm each section, using a Leica Vibratome. The sections were transferred with the help of a brush to a TC dish filled with 1 × PBS. The sections were washed with 1 × BSA for 20 min and incubated at 4 °C overnight with the primary antibodies (Desmin, Nkx2.2 and HNK1) diluted in 1 × BSA. For negative control, we omitted the primary antibody incubation step. To block the endogenous peroxidase activity and reduce the background, the sections were incubated with 0.3 H_2_O_2_ (30%) in 1 × PBS at RT for 15 min. The sections were washed with 1 × PBS for 10 min, and then incubated at RT with the secondary antibody (goat-anti-mouse IgG HRP) diluted in 1 × PBS (5:1000) for 2 h. The sections were washed two times with 1 × PBS for 10 min each. The PBS was exchanged with DAB staining solution. After removing the staining solution, the sections were washed with 1 × PBS and fixed in 4% PFA for 20 min. The sections were washed with distilled H_2_O and transferred with the help of brush to the microscope slides. Thereafter, the sections were dried and mounted with Aquatex (Merk).

For further information regarding the material and reagents required for the experiments please check the Supplementary Material.

### Microscopy and imaging

Photos were taken using Leica stereo microscope (M165 FC, Germany) equipped with a digital camera (DFC420 C, Leica, Germany) and Virtual slide microscope (Olympus, BX61VS). The photos were further processed using ACDSee software (ACD Systems) and CorelDRAW 4X (Corel Corporation). Scale bars were measured using ImageJ software.

### Ethics approval

According to German legislation, the use of embryonic vertebrates in an animal experiment needs approval only if the animal is in the last third of its embryonic development. In the case of chicken, this means that experiments done on animals before embryonic day 14 (E14) are not regarded as an animal experiment by the Tierschutzgesetz, and therefore, do not need approval or governmental permission.

## Results

### Double whole-mount ISH

**Fig. 1:**
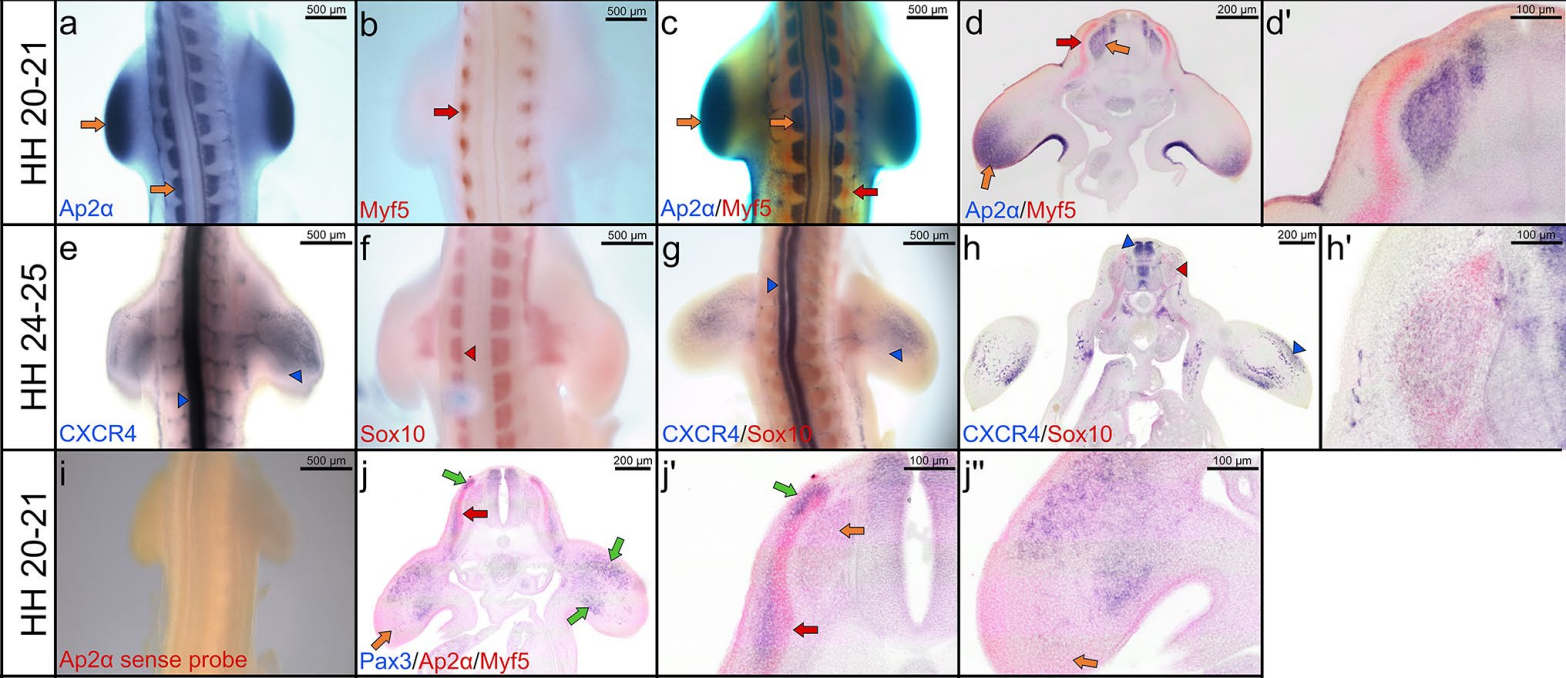
Double whole-mount ISH. Representative embryos are shown below: **a–c** Embryos of a stage HH20-21 are labelled with a DIG- Ap2α probe (blue) and a FITC-Myf5 probe (red). **d** Vibratome cross sections of double whole-mounted embryos in (**c**). **d**ʹ Higher magnification of the photo in (**d**). **e–g** Embryos of a stage HH24-25 are labelled with a DIG-CXCR4 probe (blue) and a FITC-Sox10 probe (red). **h** Vibratome cross sections of double whole-mounted embryos in (**g**). **h**ʹ Higher magnification of the photos in (**h**). **i** Chicken embryo of a stage HH22 hybridized with Ap2α sense probe as a negative control. **j** Vibratome cross sections of double whole-mounted embryos labelled with a FITC- Ap2α/ Myf5 probes (red) and a DIG-Pax3 probe (blue). Scale bar 200 μm. **j**ʹ, **j**ʹʹ Higher magnification of the photos in (**j**). Orange arrows indicate Ap2α expression; red arrows indicate Myf5 expression; blue arrowheads indicate CXCR4 expression; red arrowheads indicate Sox10 expression; green arrows indicate Pax3 expression. Whole-mount photos were taken at a magnification of 5×. Cross section photos were taken at a magnification of 40×

Double whole-mount ISH is performed within a period of 6–7 days and an example of multiple labelling is given in Fig. 1. In this figure, double whole-mount ISH is used to study the pattern of gene expression in mesodermal and neural crest cells. Chicken embryos are hybridized with DIG (Ap2α and CXCR4) and FITC (Myf5 and Sox10) riboprobes. The embryos can be hybridised simultaneously with two FITC—labelled and one or two DIG—labelled antisense RNA probes. In this case, the double whole-mount ISH protocol represents three genes (Fig. 1j, jʹ, jʹʹ). Prior to performing the double whole-mount ISH protocol, we always test our FITC and DIG probes individually using the standard ISH protocol (Fig. 1a, b, e, f). An Ap2α sense FITC-probe was used as a negative control, and no staining signals with this probe was detected (Fig. 1i). The resulting staining signals can be observed using the virtual microscope and Leica M165 FC microscope.

### Combined double in situ hybridization and immunostaining

**Fig. 2:**
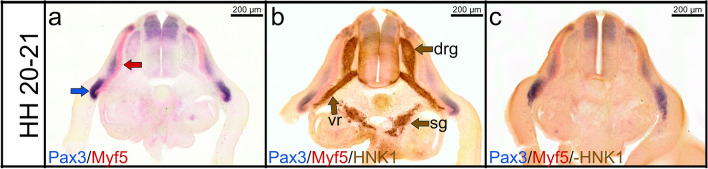
Combined double ISH and immunostaining labelling for Pax3, Myf5 and HNK1. **a** Cross section of a stage HH20-21 labelled with Pax3 probe in blue (DIG) and Myf5 probe in red (FITC). The dermomyotome is labelled with Pax3 probe in blue (blue arrow in **a**). Myf5 transcripts are expressed in the myotome (red arrow in **a**). **b** Immunostaining is performed for HNK1 on the same cross section in **a**. HNK1 is detected in dorsal root ganglia, ventral roots and sympathetic ganglia (brown arrows in **b**). **c** Immunostaining is performed as in (**b**) but without primary antibody added. Cross section photos were taken at a magnification of 40×

The combination of multicolour ISH and immunostaining enables us to simultaneously detect mRNA transcripts and their proteins product in the same section. Since the secondary antibody (HRP-conjugated goat anti-mouse) used for immunostaining may bind to mouse monoclonal anti-FITC-AP antibody used for double ISH, we omitted the primary antibody incubation step and used only HRP-conjugated goat anti-mouse secondary antibody. No immunostaining signals were observed in the negative control omission of the primary antibody (Fig. 2c). Thus, our results confirm the lack of cross-reaction between double ISH and immunostaining.

### Comparison of CXCR4, Ap2α, Sox10, HNK1 and Desmin expression

**Fig. 3:**
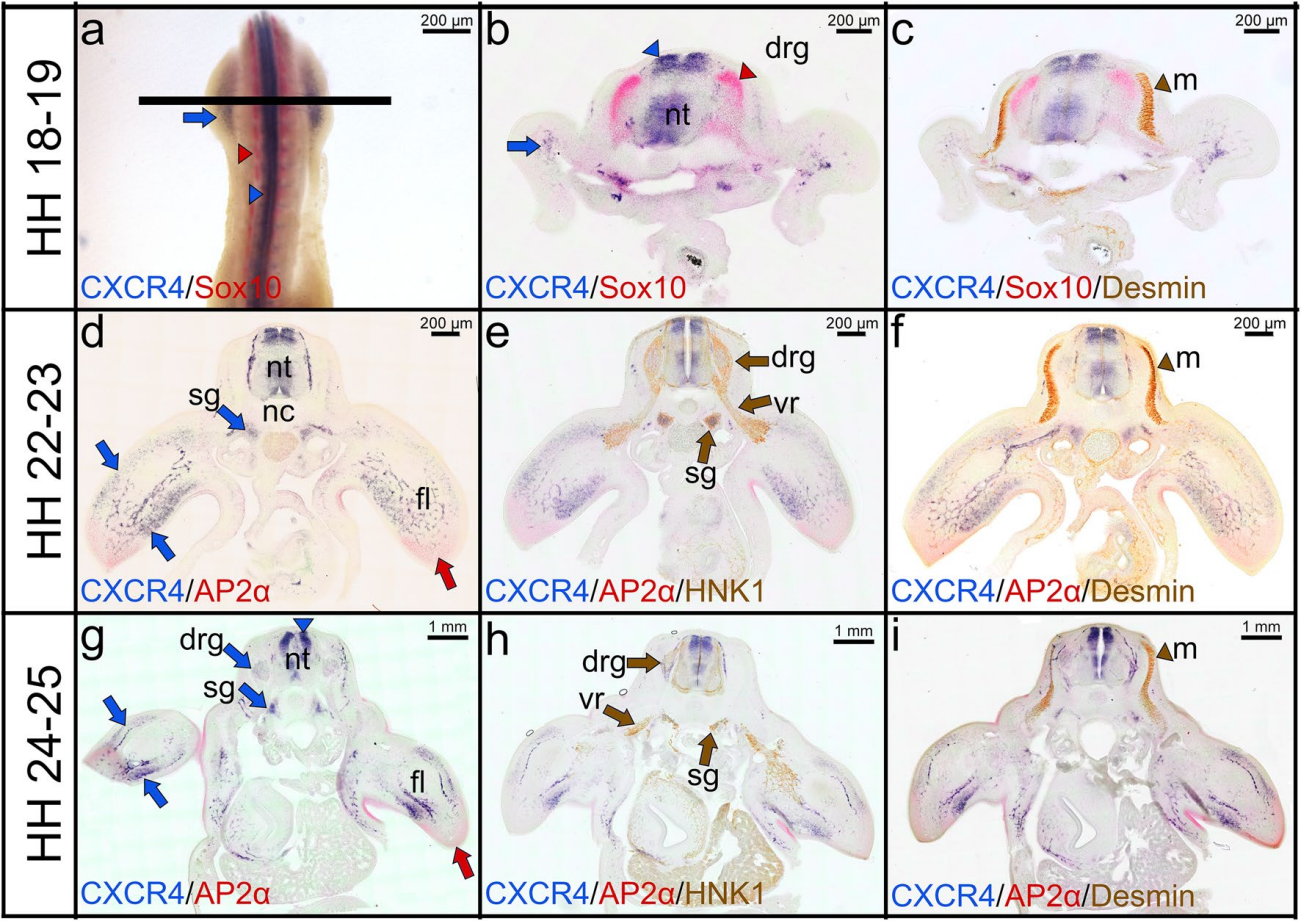
Combined double whole-mount ISH and immunostaining labelling for CXCR4, Sox10, Ap2α, HNK1 and Desmin. **a** Double whole-mounted embryo labelled with CXCR4 probe in blue (DIG), and Sox10 probe in red (FITC). CXCR4 transcripts are detected strongly in the limb bud (blue arrows) and neural tube (blue arrowhead). Sox10 transcripts are expressed in the dorsal root ganglia (red arrowhead). **b** Vibratome cross sections (indicated by the line in **a**) showing expression of CXCR4 (blue) and Sox10 (red). **c** Immunostaining was performed for Desmin on the same cross section in (**b**). **d**, **g** Vibratome cross sections showing expression of CXCR4 (blue) and Ap2α (red). **e**, **h** Immunostaining was performed for HNK1 on the same cross section in (**d**) and (**g**). **f**, **i** Immunostaining was performed for Desmin on the same cross section in (**d**) and (**g**). CXCR4 is expressed in the sympathetic ganglia (blue arrows in **d**, **g**). The myotome is labelled with Desmin antibody in brown (brown arrowheads in **f**, **i**). CXCR4 transcripts and HNK1 antibody are co-expressed in sympathetic ganglia. CXCR4 and Desmin are co-expressed in the limb buds. HNK1 is detected in dorsal root ganglia, ventral roots and sympathetic ganglia (brown arrows in **e**, **h**). Ap2α transcripts are expressed in the distal mesenchyme (red arrows) of limb buds. *fl* forelimb, *nt* neural tube, *nc* notochord, *drg* dorsal root ganglia, *vr* ventral root, *m* myotome, *sg* sympathetic ganglia. Photos are taken with a magnification 40×

**Fig. 4:**
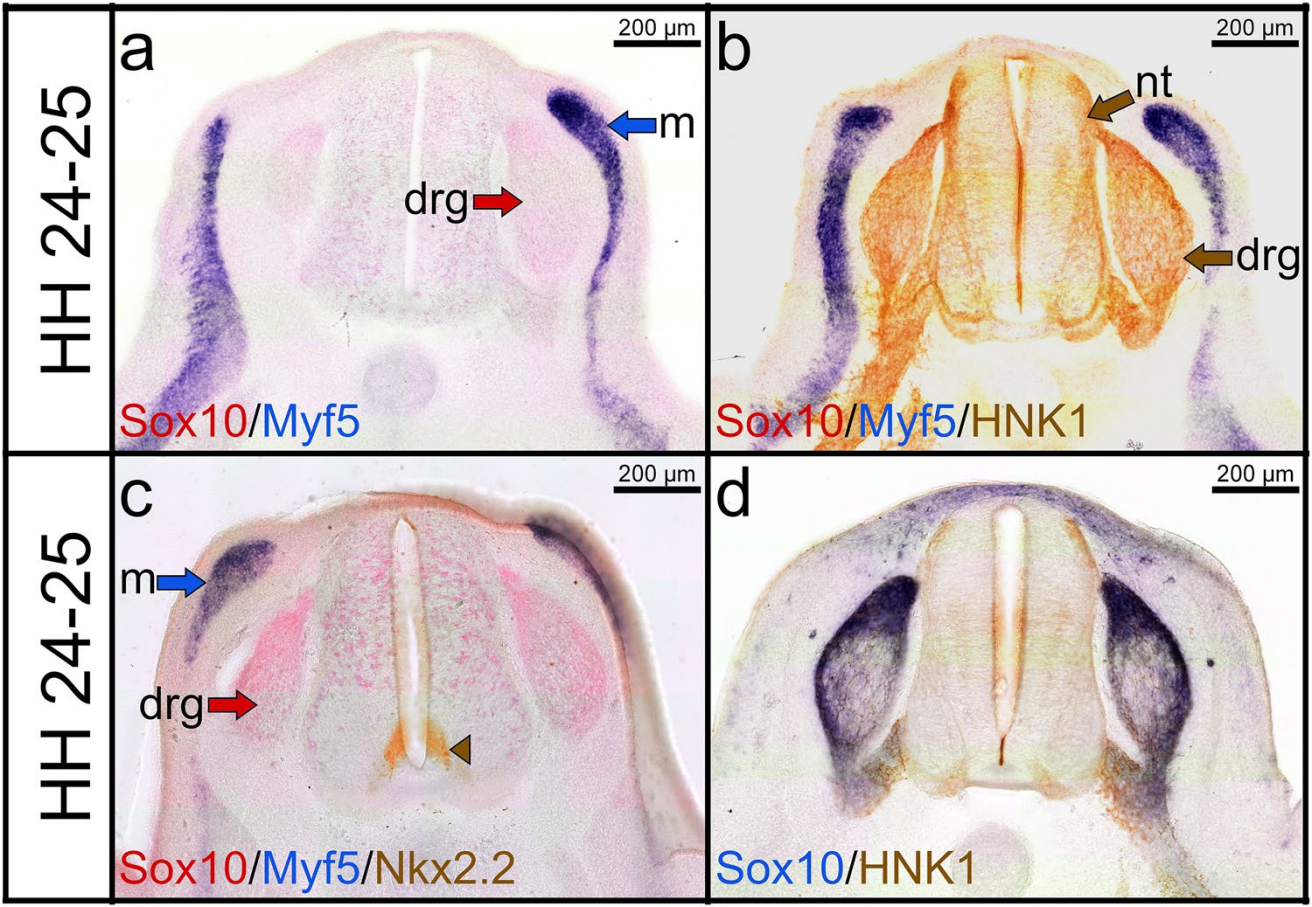
Combined double ISH and immunostaining labelling for Myf5, Sox10, HNK1 and Nkx2.2. **a** Cross section of a stage HH24-25 labelled with Myf5 probe in blue (DIG) and Sox10 probe in red (FITC). **b** Immunostaining is performed for HNK1 on the same cross section in (**a**). **c** Immunostaining was performed for Nkx2.2. **d** Cross section of the same stage labelled with Sox10 probe and stained for HNK1. HNK1 and Sox10 expression domains overlapped in the dorsal root ganglia and ventral roots. Note that Sox10 signals (red colour) are obscured by HNK1 (brown colour) signals. Ventromedial neural tube cells are labelled with Nkx2.2 (brown arrowhead in **c**)

In order to precisely define the CXCR4 expression domain in relation to the various steps of neural crest and mesodermal progenitor cells migration, we compared its expression with that of Ap2α, Sox10, HNK1 and Desmin by combined double whole-mount ISH with immunostaining during development of the forelimb. Figure 3 shows cross-sections through forelimbs stained for HNK1 and Desmin after double whole-mount ISH with CXCR4 and Sox10 probes (Fig. 2a–c) or CXCR4 and Ap2α probes (Fig. 3d–i). At stages HH18-19, CXCR4 expression is associated with the neural tube, the sympathetic ganglia and the ventral roots (Fig. 3a–c). As previously reported (Cheng et al. 2000), Sox10 expression is observed in the dorsal root ganglia and the ventral roots (Fig. 3b, c). CXCR4 is also co-expressed with HNK1 in the dorsal root and sympathetic ganglia (Fig. 3e, h). In the mesodermal lineage, CXCR4 is also detected in the limb muscle progenitor cells and angioblasts/endothelial cells (Fig. 3d, g). Expression in the neural tube remained higher at stages HH24-25, while expression in the neural crest cells only persisted in the dorsal root and sympathetic ganglia (Fig. 3d, g). Ap2α expression is detected in the distal mesenchyme of the limb buds (Fig. 3g–i). At stages HH22-23 and HH24-25, HNK1 strongly labelled the dorsal root ganglia, sympathetic ganglia and ventral roots (Fig. 3e, h). The myotome was labelled with Desmin antibody (Fig. 3f, i). Vibratome cross sections revealed a prominent staining of Sox10 (red colour) in the dorsal root ganglia at stage HH24-25 (Fig. 4a), however, the staining is obscured in these tissues after combining it with HNK1 immunostaining (brown colour) (Fig. 4b). In contrast, when the vibratome sections were labelled with Nkx2.2 antibody (marker for ventromedial neural tube cells) following double ISH, Sox10 transcripts were better visible in the dorsal root ganglia (Fig. 4c). The expression of Sox10 (red colour) was also observed in the dorsal root ganglia after HNK1 staining when the embryos hybridized with the DIG-labelled Sox10 probe (Fig. 4d).

### Relationship between mesodermal and neural crest cells during forelimb development

**Fig. 5:**
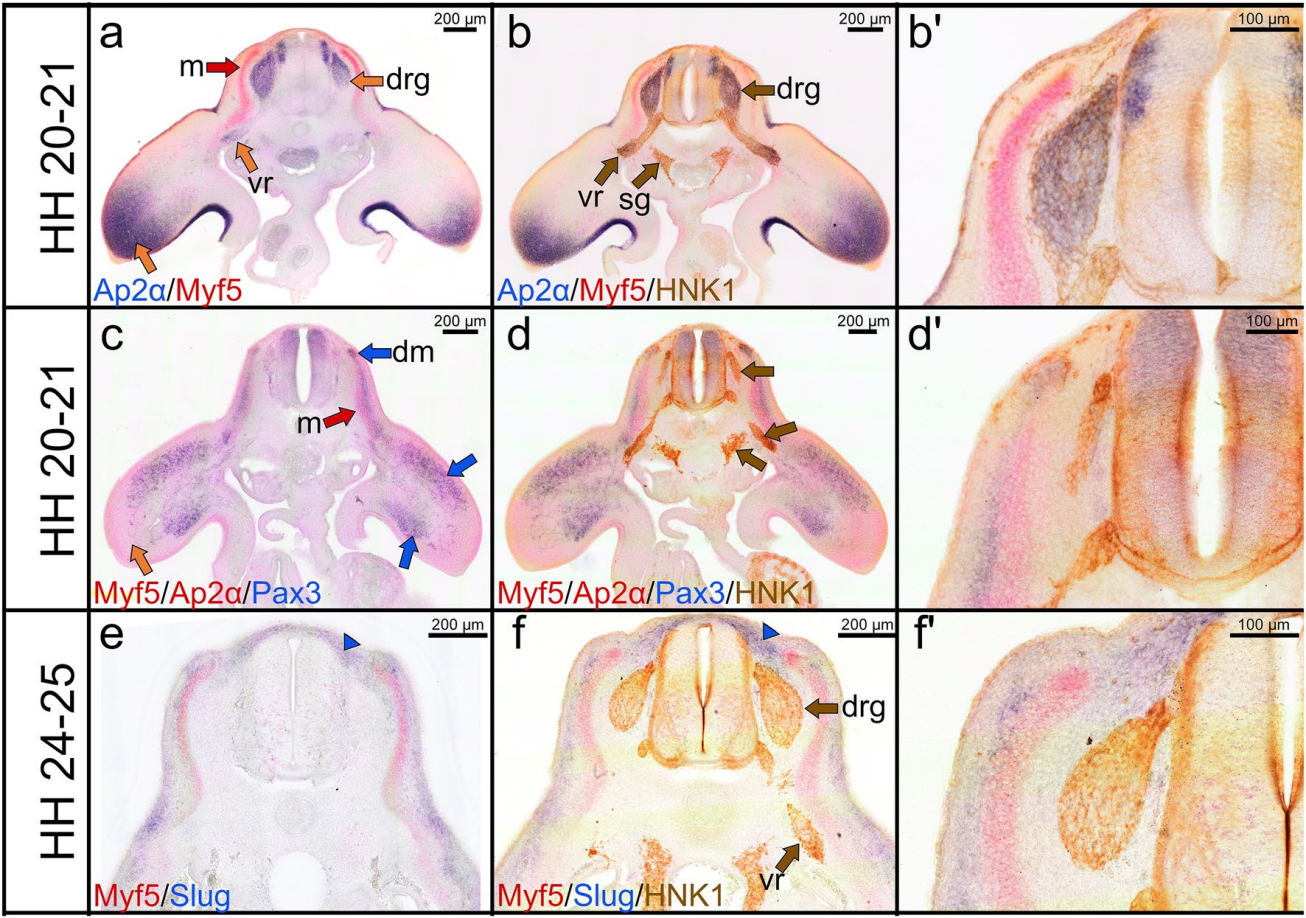
Combined double ISH and immunostaining labelling for Myf5, Ap2α, Slug, Pax3 and HNK1. **a** Cross section of a stage HH20-21 chicken embryo labelled with Ap2α probe in blue and Myf5 probe in red. **b** Immunostaining is performed for HNK1 on the same cross section as presented in (**a**). **b**ʹ Higher magnification of the photos in (**b**). Myf5 signals are found in the myotome (red arrow in **a**). Ap2α is expressed in the dorsal root ganglia, ventral roots and distal limb bud (orange arrows). The dorsal root ganglia and ventral root are co-labelled with HNK1 in brown colour (brown arrows **b**). HNK1 is faintly expressed in the neural tube (**c**) Cross section of a stage HH20-21 stained for Myf5/Ap2α in red (FITC) and Pax3 in blue (DIG). **d** Immunostaining is performed for HNK1 on the same cross section as presented in (**c**). **d**ʹ Higher magnification of the photos in (**d**). Pax3 is expressed in the dermomyotome and migrating muscle progenitor cells in the limb bud (blue arrows **c**). **e** Cross section of a stage HH24-25 stained for Myf5 in red (FITC) and Slug in blue (DIG). **f** Immunostaining is performed for HNK1 on the same cross-section as presented in (**e**). **f**ʹ Higher magnification of the photo in (**f**). Note Slug expression in the meninges surrounding the dorsal root ganglia (blue arrowheads). The abbreviations of the cross sections are as indicated before

In order to examine the expression pattern of mesodermal markers (Myf5) and neural crest markers (Ap2α, HNK1 and Sox10) during forelimb formation, we performed multi-labelling. Myf5 expression is detected in the myotome (Fig. 5a, c, e). Pax3 is expressed in the dermomyotome and migratory premuscle progenitor cells (Fig. 5c). As previously reported (Marin and Nieto 2004), Slug is expressed in the mesenchymal tissue corresponding to the prospective meninges (Fig. 5e, f). HNK1 and Ap2α are co-expressed in neural crest cells condensing to form the dorsal root ganglia and in the ventral roots (Fig. 5b, bʹ). Ap2α transcripts are observed in the distal part of the forelimbs (Fig. 5a, c).

## Discussion

### Chicken model

The chicken embryo has become steadily a more powerful research model thanks to several approaches: in vivo bead implantation and electroporation (allowing gain and loss of function), transgenesis methods, embryonic stem cells, grafting and lineage tracing (Stern 2005). Unlike in rodent models, dealing with chicken embryos does not affect the mother. Moreover, chicken eggs are easy to obtain and are an inexpensive source of biological material (Tolosa et al. 2013). It has been well documented that the chicken genome has a similar number of genes compared to humans and it represents a very high level of conserved synteny with mammals (Tolosa et al. 2013). The present method offers the advantage of the simultaneous detection of two cell populations at the chicken embryo forelimb level. Double whole-mount ISH method is used for simultaneous analysis of two or three genes at mRNA level, followed by immunostaining technique carried out on vibratome sections of the same embryo to examine the third or fourth genes at protein level.

### Double whole-mount ISH

ISH is an extremely useful research tool for identifying gene expression changes in specific cell populations in tissue sections and whole-mount samples across many research fields (Grabinski et al. 2015). DNA ISH has been optimized to detect the structure of chromosomes. While, RNA ISH can be used to determine mRNAs in whole-mounts or tissue sections samples (Lee et al. 2013). ISH probes can be directly labelled with the fluorescein (direct ISH) so that no enzymatic immunostaining visualization procedure is required. Reduction in nonspecific background staining is another significant benefit of direct method. Indirect ISH require the labelled probes to be accessible to conjugated antibodies. The most popular systems are DIG and FITC labelled probes. The detection of DIG or FITC labelled probes may be performed using high affinity antibodies that are coupled either to peroxidase, alkaline phosphatase, rhodamine, or fluorescein. Alternatively, biotin-labelled or dinitrophenol-labelled probes can be used in a similar way as DIG and FITC probes. Biotinylated probes can be detected by avidin substrate or anti-biotin antibodies. In spite of the clear and sharp localization of mRNA visualized by direct ISH technique, indirect methods are often preferred. To investigate whether a novel gene might be involved in distinct lineage specification, it is often useful to relate the position of its expression domains with those of well-characterized regional markers. The availability of biotin as a third RNA labelled probes allow to detect the expression of three genes at the ISH level using different three-color substrates. Double ISH can also be used to target three mRNAs at the same time. However, different signals locations for both double ISH probes and immunostaining markers (antibodies) are often necessary for clear visualization of the three mRNAs. In such three mRNAs ISH detection cases, whole-mount chicken embryos can be labelled with Ap2α (distal limb marker) and Myf5 (myotome marker) in red, and Pax3 (dermomyotome marker) in blue. It is also possible to follow the localization of three different mRNAs simultaneously in whole-mount ISH, using Pax1 (sclerotome marker) FITC or DIG probe in combination with Pax3-DIG/Myf5-FITC probes. For the immunostaining step, cross section corresponding to the whole-mount embryos can be stained for HNK1 (dorsal root ganglia, ventral roots and sympathetic ganglia marker) in brown. This makes whole-mount double ISH a powerful technique to follow embryological processes (e.g. cell migration). In principle, the embryos are hybridized with different riboprobes, one/two coupled to Digoxigenin (DIG), and one/two coupled to Fluorescein isothiocyanate (FITC) (Psychoyos and Finnell 2008). DIG- and FITC-labelled probes are then detected separately, since their antibodies are coupled with the same alkaline phosphatase enzyme (Dietrich et al. 1997). We normally use AP-based BCIP/NBT staining for the anti-DIG conjugate and AP-Fast Red staining for the anti-FITC conjugate. As a blue colour, instead of BCIP/NBT (Sigma) BM purple (Boehringer) can be used.

Alternatively, DIG and FITC probes can be detected with the same color. For such double-target ISH case, FITC probe can be identified by the light blue color that result from treatment with an AP-conjugated antibody against FITC and BCIP substrate. Whereas, DIG probe can be detected by dark blue color that developed from treatment with an AP-coupled anti-DIG and BM purple or NBT/BCIP substrate (Basch et al. 2006). Moreover, the visualization of two different probes using the same colour has been described by Escot et al. (2013). In this study, they labelled the section with SDF-1 in light blue and CXCR4 in dark blue. In general, Fast Red is less sensitive than NBT/BCIP substrate. The anti-FITC gives darker background, which when stained in blue can interfere with the specific staining signal (Dietrich et al. 1997). Since anti-DIG and the NBT/BCIP substrate are both more sensitive, it is crucial to use DIG labelling for the detection of transcripts present at very low levels (Brend and Holley 2009; Dietrich et al. 1997; Psychoyos and Finnell 2008). Furthermore, the protocol involving DIG-labelled probes yielded staining signals in a much shorter time (Schaerenwiemers and Gerfinmoser 1993). Therefore, we strongly suggest that DIG labelling should be used for the weaker probe.

Advances in genetic engineering have made transgenic mice very useful for analysis of gene expression and function (Schatz et al. 2005). Genetically manipulated mice expressing β-galactosidase (lacZ) or green fluorescent protein (GFP) have been widely used to monitor gene expression patterns in whole-mount or tissue sections (Kelly et al. 1995; Schatz et al. 2005). lacZ staining (also known as X-gal staining) is marked by a dark blue stain (Burn 2012). In recent years, establishment of chicken embryo xenograft and lacZ reporter models have been achieved (Cordeiro et al. 2015; McGrew et al. 2010). Study by McGrew and his colleagues suggested that the structure of the myosin light chain 1/3 locus is highly conserved between chicken and mammals. They were able to drive expression of a lacZ reporter gene in the chicken skeletal muscle (fast fibres) in a pattern similar to the endogenous myosin light chain locus (McGrew et al. 2010). This may enhance the possibility of combining lacZ staining with immunostaining.

### Immunostaining after double whole-mount ISH

As described above, ISH offers many advantages over most other methods used for identifying gene expression profiles in tissue samples. The best example of these advantages is the identification of cell populations that express target genes by combining ISH with immunohistochemistry, on the same tissue section as previously described (Chaudhuri et al. 2013; Kumar et al. 2012; Lopez 2014; Pineau et al. 2006). A variety of such multicolour immunostaining procedures have been reported with either HRP or AP, using primary antibodies raised in different animal species. Double immunostaining method for the simultaneous detection of two proteins, using HRP and AP conjugates has been previously described (Vanderloos et al. 1988). Recently, a new double immunostaining method with two HRP substrates (Magenta and DAB) has been described (Petersen et al. 2018). As substrates for HRP, in addition to Magenta and DAB either AEC (red) or HRP green may work. Alternative multicolour immunostaining detection procedures include the Avidin–Biotin complex in combination with DAB labelling. Instead of antibodies conjugates (enzyme conjugated AP or HRP), antibodies labelled with fluorescent dyes could also be used. Thus, the immunostaining methods mentioned above allow great flexibility with regard to multicolours combinations. Several commercially kits based on HRP or AP conjugated secondary antibodies exist to perform double or triple immunostaining. The possibility of double immunostaining to detect two markers is commonly reduced by using antibodies obtained from different species (Vanderloos et al. 1993). Unlike production of specific antibodies for immunostaining, species-specific RNA probes for ISH can easily be synthesized. As a consequence, development of such a method in chicken embryos became necessary because of the unavailability of some primary antibodies that work specifically in chickens, in contrast to mice. To overcome this particular problem, we have, therefore, developed an uncomplicated multiple labelling method that does not involve more than one primary antibody for immunostaining. The present method allows immunostaining on floating vibratome sections following double whole-mount ISH. The multiple labelling of a double whole-mount ISH and stained section ISH can be more readily examined in the virtual slide microscope or any light microscope.

The combination of mRNA detection by double whole-mount ISH and protein detection by the use of immunostaining is an efficient technique to identify more than two genes within the same section. However, there are some critical aspects to the method that should be taken into consideration by researchers. Since the anti-FITC conjugate and the fast-red substrate are both less sensitive, they should not be used for determining the overlap of the expression domains of two genes if the signals from the immunostaining (brown colour) are co-localized with that of the FITC probe (red colour). To overcome this problem, we recommend the use of DIG probe (blue colour) for determining the co-localization of the expression domains of mRNA transcripts with that of the protein.

In conclusion, the three color combined double whole-mount ISH with immunostaining method is used for determining both spatial and temporal patterns of gene expression or the overlap of the expression domains of two genes. Our protocol provides useful tools for tracing migratory mesodermal and neural crest cells, but this method may also be used to detect five genes in various cell types or tissues and can be adapted for use in different model organisms. Our protocol opens a variety of gene expression pattern analyses following chicken embryo manipulation (bead implantation and electroporation of DNA or RNA constructs).

## Electronic supplementary material

Below is the link to the electronic supplementary material.

Supplementary file1 (PDF 292 kb)

## Data Availability

All data generated or analysed during this study are included in this published article.
