# Supplementary material for: How to distinguish between different cell lineages sharing common markers using combinations of double in-situ-hybridization and immunostaining in avian embryos: CXCR4-positive mesodermal and neural crest-derived cells
Source: Histochem Cell Biol. 2020 Oct 10;155(1):145–55. doi: 10.1007/s00418-020-01920-7 (PMC7847855; doi:10.1007/s00418-020-01920-7)
Supplement: Supplementary file 1 — Supplementary file1 (PDF 292 kb) [file 418_2020_1920_MOESM1_ESM.pdf]

How to distinguish between different cell lineages sharing common markers using combinations of double in-situ-hybridization and immunostaining in avian embryos: CXCR4-positive mesodermal and neural crest-derived cells

Journal of Histochemistry and Cell Biology

Imadeldin Yahya[1,2], Marion Böing[1], Beate Brand-Saberi[1], Gabriela Morosan-Puopolo[1]✉

[1]Institute of Anatomy, Department of Anatomy and Molecular Embryology, Ruhr University Bochum, Germany.

[2]Department of Anatomy, Faculty of Veterinary Medicine, University of Khartoum, Sudan.

Corresponding authors: Gabriela.Morosan-Puopolo@ruhr-uni-bochum.de

Materials and Reagents

A. Chemicals required for double whole-mount ISH

1. Diethyl-pyro-carbonate (DEPC)-treatment: All auto-cleavable solutions should be made with DEPC- treated water to destroy any residual RNase.
2. 1xPBS: dissolve in 800 ml distilled H_2O (8 g NaCl, 0.2 g KCl,1.44 g Na_2HPO_4, 0.24 g of KH_2PO_4, Adjust the pH to 7.4 with HCl) adjust the volume to 1L with additional distilled H_2O, DEPC-treat and autoclave.
3. PBST: 0.1% Tween-20 in DEPC-treated and autoclaved 1xPBS.
4. 4% PFA/1x PBS: dissolve 4 g of paraformaldehyde in 50 ml prewarmed H_2O at 60°C (approx. 1 ml of 1M NaOH may be added to assist dissolving). Add 10 ml of 10x PBS for a final concentration of 1xPBS and allow the mixture to cool at room temperature.
5. Myf5, Pax3, Sox10, Slug, Ap2 and CXCR4 chicken probes.

6. 20x SSC pH 4.5: dissolve in 800 ml distilled H_2O (175.3g NaCl, 88.2g sodium citrate) adjust pH with citric acid, adjust the volume to 1L with additional distilled H_2O, DEPC-treat and autoclave.
7. Prehybridization solution: 50% formamide (highest grade), 5x SSC pH 4.5, 2% SDS, 2% Boehringer's blocking reagent (powder), 250 µg/ml tRNA (from stocks or powder), 100 µg/ml heparin, keep at -20°C.
8. KTBT: 100 mM maleic acid, 150 mM NaCl, 0.1% Tween-20, pH 7.5.
9. 10x TBST: 8g NaCl, 25 ml 1M Tris-HCl pH 7.5, 0.2 g KCl, 10 ml Tween-20 per 100 ml.
10. 10% CHAPS: dissolve 5 g CHAPS in 50 ml distilled H_2O.
11. Posthybridization buffer 1: 2 x SSC, 0.1 % CHAPS.
12. Posthybridization buffer 2: 0.2 x SSC, 0.1 % CHAPS.
13. Alkaline phosphatase buffer (AP): 2 ml NaCl (5M), 5 ml MgCl2 (1M), 10 ml Tris (1M), pH 9.5, 1 ml Triton-x-100 (10 %), Add H2O bidest sterile to a final volume of 100 ml.

B. Chemicals required for immunostaining

1. Double distilled H_2O, 1x PBS and 4% PFA in PBS.
2. 1x BSA: Bovine Serum Albumin 1g in 100 ml 1xPBS.
3. Staining solution: 250 µl DAB (0.16 mg/ml), 1750 µL PBS, 2 µl of 30 %H_2O_2.
4. Mouse anti-Chicken HNK1 antibody (Hybridoma Bank), Mouse anti- desmin antibody (from Dako) and Goat anti-mouse IgG HRP (Dako).
5. Agarose (Serva).
6. Mounting media (Merck) and Pattex glue
